# A structured review and exploration of the healthcare costs associated with stillbirth and a subsequent pregnancy in England and Wales

**DOI:** 10.1186/1471-2393-13-236

**Published:** 2013-12-17

**Authors:** Hema Mistry, Alexander E P Heazell, Oluwaseyi Vincent, Tracy Roberts

**Affiliations:** 1 Health Economics Unit, School of Health and Population Sciences, University of Birmingham, Edgbaston, Birmingham B15 2TT, UK; 2 Manchester Academic Health Science Centre, St Mary’s Hospital, Oxford Road, Manchester M13 9WL, UK; 3 York Health Economics Consortium, University of York, York YO10 5NH, UK

**Keywords:** Costs and cost analysis, Stillbirth, Multiparous women, NHS

## Abstract

**Background:**

In contrast to other pregnancy complications the economic impact of stillbirth is poorly understood. We aimed to carry out a preliminary exploration of the healthcare costs of stillbirth from the time of pregnancy loss and the period afterwards; also to explore and include the impact of a previous stillbirth on the healthcare costs of the next pregnancy.

**Methods:**

A structured review of the literature including cost studies and description of costs to health-care providers for care provided at the time of stillbirth and in a subsequent pregnancy. Costs in a subsequent pregnancy were compared in three alternative models of care for multiparous women developed from national guidelines and expert opinion: i) “low risk” women who had a live birth, ii) “high risk” women who had a live birth and iii) women with a previous stillbirth.

**Results:**

The costs to the National Health Service (NHS) for investigation immediately following stillbirth ranged from £1,242 (core recommended investigations) to £1,804 (comprehensive investigation). The costs in the next pregnancy following a stillbirth ranged from £2,147 (low-risk woman with a previous healthy child) to £3,751 (Woman with a previous stillbirth of unknown cause). The cost in the next pregnancy following a stillbirth due to a known recurrent or an unknown cause is almost £500 greater than the pregnancy following a stillbirth due to a known non-recurrent cause.

**Conclusions:**

The study has highlighted the paucity of evidence regarding economic issues surrounding stillbirth. Women who have experienced a previous stillbirth are likely to utilise more health care services in their next pregnancy particularly where no cause is found. Every effort should be made to determine the cause of stillbirth to reduce the overall cost to the NHS. The cost associated with identifying the cause of stillbirth could offset the costs of care in the next pregnancy. Future research should concentrate on robust studies looking into the wider economic impact of stillbirth.

## Background

In the UK, stillbirth is defined as “a baby delivered with no signs of life, known to have died at 24 completed weeks of pregnancy onwards” [1]. In 2012, there were 3,558 stillbirths in England and Wales, a rate of 4.9 per 1,000 total births [2]. Despite advances in obstetric care, the incidence of stillbirth is not significantly lower than 25 years ago (1987: 3,423 stillbirths, a rate of 5.0 per 1,000 total births). Of concern is the UK stillbirth rate which ranks 33rd out 35 high-income countries [2,3]. In contrast to other pregnancy complications such as preterm birth and preeclampsia [4-6], the economic impact of stillbirth is poorly understood. This is problematic for two reasons; first it underestimates the societal impact of stillbirth in comparison to other pregnancy complications. Second, it represents a major obstacle in the evaluation of strategies to reduce stillbirth, as investigators cannot compare the cost of an intervention against the reduction in costs associated with stillbirth. Recently, several reports have discussed issues related to health economics of stillbirth [7-9].

Potential economic costs associated with stillbirth may be grouped temporally, into i) those which occur at the time of initial management of the stillbirth, ii) those which are incurred after the initial management has been completed and iii) those which occur specifically in a subsequent pregnancy. Clinical guidance from the Royal College of Obstetricians and Gynaecologists (RCOG) recommends that pregnancy after stillbirth is managed as high-risk [10], this is due to the significantly elevated risk of a stillbirth in a subsequent pregnancy [11]. However, this does not apply to all causes of stillbirths, some of which result from non-recurrent events such as viral infection, isolated structural fetal anomaly or umbilical cord accident [12]. Stillbirths, associated with placental complications such as abruption and fetal growth restriction (FGR) are associated with a two to ten-fold higher recurrence risk of stillbirth in the next pregnancy [13]. In a recent study of 163 women, 11 (6.7%) had a subsequent stillbirth, of which at least 6 (54%) had similar pathologies in both events [14]. To address the increased risk of recurrent stillbirth, serial ultrasound scans are recommended in the next pregnancy to identify FGR and to provide reassurance to women and their partners [15]. In addition, due to increased maternal anxiety in a subsequent pregnancy some women will seek more frequent but unnecessary antenatal visits and testing, usually by ultrasound scanning, assuming, perhaps mistakenly, that they have a higher than average risk of recurrence, while other women who truly are ‘high risk’ are the ones who should be offered the extra visits and testing [16]. A better understanding of the costs to society and the health care sector, associated with stillbirth from the time of loss onwards would allow those responsible for care provision to appreciate the breadth of the impact of stillbirth and ensure that resources are used as efficiently as possible. Given the enormity of assessing the potential impacts of stillbirth we have deliberately restricted this initial assessment to the perspective of the health care provider. Therefore, we aimed to i) Review published literature to identify studies of the economic impact of stillbirth, ii) calculate the economic costs of care at the time of stillbirth including investigation of the cause, iii) calculate the economic costs of care in a subsequent pregnancy after stillbirth and compare this to women who have had a live birth and iv) consider the trade-off that exists between the cost of investigations to determine the cause of stillbirth and the potential saving of additional investigations in the next pregnancy.

## Methods

### Structured review - search strategy

A structured approach to reviewing the economic literature was undertaken using established methods [17]. Typically because of the heterogeneity of the relevant economic literature, unlike clinical trials, such reviews rely on a qualitative critique of the relevant studies as opposed to a meta-analysis. The following databases were searched: MEDLINE, EMBASE, CINAHL, Science Citation Index (SCI) and Social Science Citation Index (SSCI) by OV. In addition, reference lists of retrieved studies were also scanned. Publications relating to the cost of stillbirth were identified by combining the stillbirth terms with the economic terms an example of a search strategy is shown below. Studies which described resource use and costs (in monetary terms) associated with stillbirth were included. Studies were excluded if it was not possible to differentiate between stillbirths and other forms of pregnancy loss or child death e.g. first trimester miscarriage or if resource use was not quantified. Searching was restricted to articles published in English language and to humans, between January 1998 to October 2013.

1. stillbirth/

2. stillbirth$.mp.

3. fetal death/

4. perinatal loss.mp.

5. pregnancy loss.mp.

6. perinatal death.mp.

7. or/1-6

8. exp “costs and cost analysis”/

9. exp health care costs/

10. economics/

11. budget$.tw.

12. or/8-11

13. 7 and 12

14. limit 13 to (english language and humans)

15. limit 14 to last 10 years

A two-stage screening process was used to select papers for the review [17]. Using this strategy papers identified using the search strategy were initially categorised by one author (OV) based on titles and abstracts: A) the study reports primary or secondary research on estimating the resource use or costs associated with stillbirth; B) the study reports resource use in care pathways for women who have experienced a stillbirth; C) the study may prove to have useful information but does not fall into (A) or (B); or D) the study does not have any relevance. Any studies in (A), (B) and (C) were considered potentially relevant to this structured review. Final categorization of studies was based on reading of the full papers by two authors (OV and HM) in groups (A), (B) and (C), identified studies were included or re-classified as appropriate. All relevant cost and resource use data was then extracted from papers included in the review. Cost data in local currencies was inflated to 2010 prices using the Gross Domestic Product deflators [18] and then converted to UK £ sterling 2010 using purchasing power parities [19].

### Identification of care pathways for women immediately following a stillbirth

We explored UK guidelines from the RCOG to establish the full range of investigations that are likely to be offered to women immediately after they experience a stillbirth [10]. We compiled a list of tests, interventions and procedures based on these guidelines and used data from local perinatal audit [20] to confirm the most likely package of care for women in the UK. Care was grouped according to the RCOG guideline into items recommended for the majority cases of stillbirth (autopsy/post-mortem, placental pathology, Kleihaur test, bereavement counselling) and those recommended dependent upon the clinical presentation (biochemical, haematological, immunological and microbiological tests).

### Determining care pathways and patient numbers for pregnancy following a stillbirth

We developed care pathways for women experiencing pregnancy after stillbirth using published guidance from the RCOG, National Institute of Health and Clinical Excellence (NICE) and expert opinion (Keelin O’Donoghue, Cork, Ireland; Alexander Heazell, Manchester, UK) [10,21]. For comparison we also developed care pathways for two other cohorts of pregnant multiparous women with singleton pregnancies, i) healthy women who had an uncomplicated first pregnancy ending in a live-birth and ii) women with designated “high-risk pregnancies” due to pre-existing conditions, for example, diabetes or hypertension whose first pregnancy ended in a live birth. Pre-existing diabetes and hypertension were chosen to represent “high-risk pregnancies” because they are the most frequently encountered maternal medical disorders in clinical practice that confer a high risk status and there are published guidelines for their management during pregnancy [22,23]. To address whether determination of the cause of stillbirth altered the costs of care in a subsequent pregnancy, the group of women with previous stillbirth were further divided into three groups: (i) a known non-recurrent cause; (ii) those with a known recurrent cause; and (iii) those with an unknown cause. Therefore, we defined six groups of women overall, who were differentiated by whether or not the previous pregnancy resulted in a live birth or a stillbirth; whether the women had diabetes or hypertension (examples of ‘high-risk’ pregnancies); and whether or not the causes of the stillbirth was known.

•Group 1 - Healthy multiparous women who had an uncomplicated pregnancy (‘normal’ women) [21]

•Group 2 - High-risk multiparous women with a previous healthy child

○ Group 2a - ‘Diabetic’ women [23]

○ Group 2b - ‘Hypertensive’ women [22]

•Group 3 - Multiparous women with a previous stillbirth

○ Group 3a - Women with known non-recurrent causes (e.g. parvovirus, isolated structural fetal anomaly, umbilical cord accident which are unlikely to recur in a subsequent pregnancy)

○ Group 3b - Women with known recurrent causes (e.g. fetal growth restriction which are known to recur in subsequent pregnancies)

○ Group 3c - Women with unknown causes

Using data from published guidelines and expert opinion (as above), the number of antenatal visits and ultrasound scans were estimated for each of the different care pathways. For all groups (except group 1), pre-conception care was also included, which included at least one GP visit and one visit to a physician. For those women who had a stillbirth in a previous pregnancy, 80% of women had a term delivery of their next pregnancy (37–40 weeks gestation) and the remaining 20% were preterm (<37 weeks gestation) [24]. The preterm birth rate was assumed to be between 8 and 10% for women who had a previous healthy child [25].

With the exception of the costs of bereavement counselling, the costs for investigations following a stillbirth were obtained by one author (AH) who leads a specialist clinical service for parents following stillbirth. Unit costs for antenatal care and delivery care were obtained from NHS reference costs [26] and staff time was estimated from published salary scales [27] (see Table 1). Having established the number of antenatal visits (including ultrasound scans) and the mode of delivery for each care pathway, a mean total cost per patient for each care pathway was generated using average unit costs. All unit costs are presented in £ sterling 2010 prices and a NHS perspective was adopted. Costs incurred in the next pregnancy were not discounted because it was assumed that conception occurred within a year of the stillbirth [28]. We estimated average cost for each pregnancy pathway and a best scenario (using lower quartile costs) and a worst scenario (using upper quartile costs).

**Table 1:** Unit costs in 2010 prices

**Resource use**	**Unit costs in £s in 2009/2010 prices**			**Source**
	**Average**	**Lower quartile**	**Upper quartile**	
***Pre-conception care***				
GP visit (clinic consultation lasting 17.2 minutes)	£53	n/a	n/a	22
Physician visit (consultation lasting 25 minutes)	£45	n/a	n/a	22
Consultant led multiprofessional consultation (first attendance)	£179	£153	£205	21
Consultant led multiprofessional consultation (follow-up attendance)	£134	£111	£140	21
***Antenatal care***				
Midwife appointment (lasting 20 minutes)	£23	n/a	n/a	22
*Antenatal attendances*				
Consultant led consultant consultation (first attendance)	£150	398	£183	21
Consultant led consultant consultation (follow-up attendance)	£96	£62	£114	21
Consultant led consultant multiprofessional consultation (follow-up attendance)	£134	£111	£140	21
Consultant led midwife consultation (first attendance)	£77	£51	£86	21
Consultant led midwife consultation (follow-up attendance)	£51	£32	£58	21
Consultant led midwife multiprofessional consultation (first attendance)	£72	£72	£72	21
Consultant led midwife multiprofessional consultation (follow-up attendance)	£51	£51	£51	21
Outpatient procedure: antenatal investigation	£127	£88	£151	21
*Antenatal ultrasounds*				
Non-consultant led antenatal ultrasound (first attendance)	£63	£45	£87	21
Non-consultant led antenatal ultrasound (follow-up attendance)	£52	£40	£58	21
***Delivery care (Elective episodes)***				
Normal delivery with CC	£1,558	£893	£2,037	21
Normal delivery without CC	£1,151	£433	£1,644*	21
Assisted delivery without CC	£1,374	£1,167	£1,872*	21
Planned lower uterine caesarean section	£1,822	£1,276	£1,985	21
Emergency or upper uterine caesarean section	£2,979	£2,915	£3,463	21

*The upper quartile cost was lower than the mean cost for both cases, therefore the mean cost for non-elective was used. The costs for the previous financial year were not in line with these costs.

## Results

### Structured literature review

#### Overview of the results

Six hundred and fifty one papers were identified in the literature search after removing duplicates. No further papers were identified through the references of retrieved papers; however, one further paper was identified by one of the authors (AH); this paper was not available via any of the databases which were searched. Following review of the titles and abstracts of identified papers, it was clear that none of the studies reported information on the overall resource use or cost associated with stillbirth defined as group (A), or on the care pathways for women after a stillbirth defined as group (B). Only five papers were considered to include potentially relevant data and referred to as Group (C), and these were retrieved and the full manuscript reviewed. Three of these studies were considered to report information useful to the review. The other two studies were excluded: one was an abstract summary of one the included papers (Michalski et al., 2002) [29,30]; and the other study did not report frequency or numbers for resource use information [16]. All three studies reported information on direct costs associated with stillbirth. Two studies looked at cost of management after a stillbirth, specifically diagnostic investigations [29,31] and the other study focussed on the damages paid on stillbirth claims [30].

### Further details of the relevant studies

Michalski et al. conducted a cost-consequences analysis of comprehensive stillbirth (defined as fetal death after 20 weeks gestation) assessment [29]. Data on 1,477 stillborn pregnancies were obtained from the Wisconsin Stillbirth Service Program (WiSSP). A step-by-step costing process was carried for the stillbirth assessment, using estimates of local salaries (measures of time and wage) and material costs data. The authors estimate of the total cost of stillbirth assessment was approximately $1,450 (reported in 2002 prices), which included the cost of pathologic evaluation, cytogenetic evaluation, photographs and radiographs and other evaluations, diagnostic interpretation and counselling, plus overhead costs (estimate 45.5%) were added. Gold et al. conducted a review of patient records between 1996 and 2006 of 533 stillbirths matched with 1,053 live births in three hospitals in Michigan [31]. They calculated healthcare cost including labour, birth and any fetal testing or monitoring. The mean hospital cost for a stillbirth was $7,495 (Range: $659-$77,080) compared to $6,600 for live births (Range: $269-$64,010). Using a logistic-regression model, Gold et al. demonstrated that costs for stillbirth were greater than live births even when multiple births and serious medical complications were excluded. Mead reported information on the NHS costs of litigation and damages paid out on a 100 stillbirth claims filed between 2003 and 2007 [32]. A total of £1,761,638 was paid in damages for 62 cases, averaging £28,413 per successful claim.

### Costs of investigation and care following a stillbirth

Table 2 presents a summary of the key components for comprehensive care at the time of stillbirth; thus these are the costs of ideal care offered to parents following a stillbirth. For each woman an initial counselling session with the midwife and eight additional sessions of counselling with a specialist bereavement counsellor were assumed based on data from published sources [33,34]. We also include the cost of the post-mortem examination (this is the most costly component at £650) and the core investigations which would total £1,242. If the women had other investigations depending on the clinical scenario this could add anything from £6 to £562 to the total cost of stillbirth care. This suggests that the cost per pregnancy immediately following a stillbirth could be as high as £1,804. We found no evidence to quantify the costs in the time period between completion of the care recommended following a stillbirth and the initiation of care for a subsequent pregnancy, or to help estimate the costs which are incurred during this period.

**Table 2:** Costs of comprehensive stillbirth care‡ (UK Sterling, price year 2010)

**Resource type**	**Cost**	**Reference**
*Core investigations and counselling*		
Bereavement counselling*	£521	[16,22]
Autopsy/post-mortem	£650	University of Manchester Hospital Laboratory
Placental pathology	£54	University of Manchester Hospital Laboratory
Kleihauer test	£17	University of Manchester Hospital Laboratory
***Total core investigations***	***£1,242***	
*Other investigations (depending on clinical scenarios)*		
Cytogenetics	£246	University of Manchester Hospital Laboratory
Thrombophilia screen	£132	University of Manchester Hospital Laboratory
*- Prothrombin gene variant, Factor V Leiden mutation, Antithrombin III, Protein C, Protein S, Lupus anticoagulant, anticardiolipin antibodies.*		
Pre-eclampsia	£20	University of Manchester Hospital Laboratory
*- Urea and electrolytes, liver function tests, serum urate.*		
HbA1c	£6	University of Manchester Hospital Laboratory
Haematology	£24	University of Manchester Hospital Laboratory
*- Full blood count, DIC screen*		
Immunology	£9	University of Manchester Hospital Laboratory
Biochemistry	£28	University of Manchester Hospital Laboratory
*- Bile acids, thyroid function tests, C-reactive protein.*		
Microbiology	£55	University of Manchester Hospital Laboratory
*- Blood cultures, mid-stream urine, vaginal swabs, cervical swabs.*		
Serology	£42	University of Manchester Hospital Laboratory
*- Parvovirus B19, rubella (if nonimmune at booking), CMV, herpes simplex and Toxoplasma gondii*		
***Total cost for all tests***	***£1,804***	

*Initial session with midwife plus 8 sessions with a specialist bereavement counsellor.

‡ The resource use for comprehensive stillbirth care were obtained from RCOG guidelines on late intrauterine fetal death and stillbirth [10]. The guidelines recommend that all women should be offered full post-mortem examination which would include an autopsy, x-rays and pathological examination of the cord and placenta. In addition they suggest a list of diagnostic tests, these include the core tests recommended for all women, such as the Kleihauer test. Other investigations are recommended based on the clinical scenario such as maternal haematology and biochemistry tests used to investigate pre-eclampsia and multi-organ failure.

### Antenatal and delivery care costs in a subsequent pregnancy

Table 3 shows the frequency of antenatal attendances and ultrasound scans for each of the care pathways and Table 4 shows the probable modes of delivery for each care pathway based upon data from perinatal audit and population-based studies of mode of delivery [35-37]. The costs for subsequent pregnancy in the individual groups are shown in Tables 3 and 5. For those women who delivered to full term, the cost of antenatal care ranged from £724 (Group 1 – Healthy multiparous women who had an uncomplicated pregnancy) to £2,002 (Group 3c – Women with a previous stillbirth with an unknown cause), where group 1 had 9 antenatal visits and 2 ultrasound scans compared with group 3c who had 15 antenatal visits and 5 ultrasound scans. Antenatal care costs in the next pregnancy, for a woman who had a stillbirth due to either known recurrent or unknown cause are calculated to be almost £500 more costly than for a woman who experienced a stillbirth due to known non-recurrent cause.

**Table 3:** Frequency and total numbers of antenatal attendances, investigations and ultrasound scans*

**Group**	**Pre-conception care costs**	**Total antenatal attendances**		**Ultrasound scans**		**Antenatal care**		**Total care (including antenatal and delivery care)**
		**Women who deliver premature (<37 weeks gestation)**	**Women who deliver to full term (37 to 40 weeks gestation)**	**Women who deliver premature (<37 weeks gestation)**	**Women who deliver to full term (37 to 40 weeks gestation)**	**Women who deliver premature (<37 weeks gestation)**	**Women who deliver to full term (37 to 40 weeks gestation)**	
1: Women with a previous healthy child	✗	8	9	2	2	£673	£724	£2,147
2a: Women with a previous healthy child but has diabetes	£500	11	13	5	5	£1,309	£1,411	£3,870
2b: Women with a previous healthy child but has hypertension	£188	10	12	5	5	£1,154	£1,256	£2,920
3a: Women with a previous stillbirth – known causes (non-recurrent)	£159	10	12	3	3	£1,294	£1,486	£3,235
3b: Women with a previous stillbirth – known causes (recurrent)^#^	£159	13	15	5	5	£1,779	£1,970	£3,720
3c: Women with a previous stillbirth – unknown causes^#^	£159	13	15	5	5	£1,810	£2,002	£3,751

*Antenatal ultrasound scans are reported separately from antenatal appointments, as they are usually conducted in two different departments (even though they may be conducted during the same visit to the hospital). ^#^These data are estimates based on a study that investigated antenatal visits and scans which recorded a median of 10 (range 1 to 22) visits and 6 (range 1 to 22) ultrasound scans.

**Table 4:** Probabilities for the different delivery modes for each care pathway

**Group**	**Normal without complications**	**Normal with complications**	**Assisted delivery**	**Caesarean section (emergency)**	**Caesarean section (elective)**	**Source**
1: Women with a previous healthy child	0.670	0.000	0.110	0.130	0.090	43
2a: Women with a previous healthy child but has diabetes	0.244	0.005	0.077	0.376	0.298	43
2b: Women with a previous healthy child but has hypertension	0.559	0.050	0.155	0.132	0.104	43-44
3: Women with a previous stillbirth	0.536	0.000	0.132	0.137	0.195	45

**Table 5:** Total costs of pregnancy to the NHS for all pregnant multiparous women and mean costs of pregnancy per pregnant multiparous women (UK Sterling, price year 2010)

**Group**	**Average unit costs**			**Best case scenario (using lower quartile costs)**			**Worst case scenario (using upper quartile costs)**		
	**Total costs of pregnancy**		**Mean cost per pregnant woman**	**Total costs of pregnancy**		**Mean cost per pregnant woman**	**Total costs of pregnancy**		**Mean cost per pregnant woman**
	** Number**	** %**		** Number**	** %**		** Number**	** %**	
1: Women with a previous healthy child	£812,436,000	87.4	£2,147	£498,068,000	84.6	£1,316	£962,903,000	87.6	£2,544
2a: Women with a previous healthy child but has diabetes	£80,470,000	8.7	£3,870	£65,128,000	11.1	£3,132	£92,229,000	8.4	£4,436
2b: Women with a previous healthy child but has hypertension	£36,430,000	3.9	£2,920	£25,850,000	4.4	£2,072	£43,927,000	4.0	£3,521
***Total***	***£929,336,000***	***98.4***	***-***	***£589,046,000***	***98.2***	***-***	***£1,099,059,000***	***98.4***	***-***
3a: Women with a previous stillbirth – known causes (non-recurrent)	£2,719,000	18.0	£3,235	£1,902,000	17.8	£2,263	£3,309,000	18.0	£3,937
3b: Women with a previous stillbirth – known causes (recurrent)	£9,420,000	62.2	£3,720	£6,660,000	61.9	£2,607	£11,410,000	62.2	£4,506
3c: Women with a previous stillbirth – unknown causes	£2,371,000	15.7	£3,751	£1,664,000	15.6	£2,633	£2,872,000	15.7	£4,543
***Total***	***£14,510,000***	***1.5***	***-***	***£10, 226,000***	***1.7***	***-***	***£17,591,000***	***1.6***	***-***
***Overall Total***	**£943,846,000**	**95.9**	**-**	**£599,272,000**	**95.3**	**-**	**£1,116,650,000**	**95.9**	**-**

Group 3 refers to low risk multiparous women with a previous still birth. High risk women who have a previous still birth include those suffering from diabetes and hypertension and these account for 3.1% and 1% of the population respectively of women who have a still birth and thus make up the remaining 4.1%. (i.e. 95.9% +4.1% = 100%).

In the absence of data to the contrary, we have assumed that for women who had experienced a stillbirth, the probabilities for the different modes of delivery would not differ for each of the different care pathways. When delivery costs where included, the total costs of care ranged from £2,147 (Group 1) to £3,870 (Group 2 – “High Risk” – Diabetic Multiparous Women). The cost of care in a subsequent pregnancy for women with previous stillbirth (£3,235-3,751) was comparable to other “high-risk” pregnancies (£2,920-£3,870).

Table 5 includes a summary of the main results with best and worst scenarios for costs presented on the basis of interquartile ranges. The table shows the mean cost of pregnancy to the NHS per pregnant multiparous woman and the total cost of pregnancy to the NHS for all pregnant women who followed each care pathway.

## Discussion

Our structured review highlighted a dearth of evidence associated with the economic impact of a stillbirth. We initially aimed to holistically describe the economic impact of stillbirth. However, following the structured review it was clear there were insufficient data to address this aim. Therefore, we have attempted here to quantify the healthcare costs associated with stillbirth in three time periods, i) those which occur at the time of initial management of the stillbirth, ii) those which are incurred after the initial management has been completed and iii) those which occur specifically in a subsequent pregnancy. Based on published UK guidelines, the estimated costs to the NHS for period i) investigation and care following a stillbirth ranged from £1,242 to £1,804. Given a post-mortem rate of 40% [38], this implies the overall costs of the care provided for women at the time of stillbirth is almost £6 million per annum in England and Wales (Table 5). It was not possible to estimate the costs to the NHS or to women and their families after the initial management of stillbirth had been completed until the next pregnancy (period ii) because of a paucity of evidence about the length of this period and events occurring within this period. However, we have attempted to quantify the costs of a subsequent pregnancy following a stillbirth in a bottom-up manner [39]. The cost of the care pathways and delivery ranged from £2,147 for low-risk multiparous women with a previous healthy child to £3,751 for a woman with a previous stillbirth of unknown cause. We estimate that the cost of a second pregnancy for otherwise healthy women with a previous stillbirth with a known non-recurrent cause is £1,088 greater than that for a low-risk counterpart with a previous live birth. Our preliminary analysis shows that if the cause of stillbirth is unknown, the next pregnancy would cost the NHS approximately the same as a ‘high-risk’ pregnancy such as the case of pre-existing diabetes (Unknown stillbirth £3,720 vs. Pre-existing diabetes £3,870).

If the cause of stillbirth is determined as non-recurrent, this is likely to result in lower anxiety and stress to the woman and her family and lower costs for the NHS. In contrast, additional investigations such as more frequent ultrasound scans, may be of greater benefit to women with a previous stillbirth of known recurrent cause (e.g. FGR), allowing limited resources to be prioritised to those most likely to benefit from them [40]. Consequently, performing investigations which at least exclude recurrent conditions and reduce unexplained stillbirths may result in a reduction in healthcare costs in a subsequent pregnancy. Since autopsy with placental histopathology is most likely to provide information to reduce unexplained stillbirths (changes primary diagnosis in 9-34%, confirms diagnosis in 49-54%) these data provide additional impetus to increase the autopsy rate in the UK from the current nadir of 40% to higher levels (~55%) prior to 2000 [41].

In addition to its physical consequences, stillbirth is a profoundly traumatic experience for parents which poses the risk of long-term negative effects on the family [42] with the child’s siblings and grandparents also be emotionally affected by stillbirth [43]. The potential adverse emotional and psychological outcomes after a stillbirth may impact upon employment and relationship breakdown [44]. Considering the psychosocial consequences which may result from a stillbirth in isolation, Cacciatore suggests that the aggregate cost to society would be significant [45]. However, at present there is no quantifiable estimate for these costs to add to our analysis. To fully describe the economic implication of stillbirth, accurate estimates of these adverse psychological and social outcomes of stillbirth are required.

The main strength of this study is that it is the first which has attempted to quantify the costs associated with stillbirth and as a consequence of conducting a structured literature review it has identified an important dearth in the current evidence and knowledge on this issue. Currently the only evidence that exists that can be used is available from reference costs and the assumptions which have been explained. Using this approach this study has highlighted crucial gaps in the evidence, most notably of the economic impact of stillbirth above and beyond healthcare costs incurred in the aftercare of a stillbirth and in any subsequent pregnancies. The use of model care pathways was strengthened by the use of NICE guidelines [21-23] and from two groups’ detailed review of care in subsequent pregnancies (Manchester, UK and Cork, Ireland).

However, there are many limitations which are directly related to the paucity of evidence and the lack of data. For instance, the assumptions made that the next pregnancy post stillbirth would occur within a year and the delivery would be normal. Anecdotal evidence suggests reaching term and achieving a normal delivery are less likely for this group of women and it is likely that a high proportion of these women will be delivered early, and by caesarean due to maternal anxiety and request. If stillbirth prevention involves preterm delivery or operative delivery, there would be further additional cost implications. Thus, the direct costs to the NHS estimated in this study are, at best, conservative estimates and further studies are needed to address specific gaps in the data about subsequent pregnancy after stillbirth specifically: the mode of delivery, frequency of induction of labour or elective Caesarean birth, amount of additional antenatal contact e.g. use of out of hours services. From an international perspectives, guidelines in other countries such as Australia, New Zealand, and North America are similar to the UK [46,47]. However, pathways would have to be reanalysed in their own currencies and this may have different cost implications.

Furthermore, due to lack of evidence we have not here even attempted to calculate psychosocial costs of stress, anxiety and counselling, or the societal impact of time absent from work and employment costs. Compared to parents who have a live birth, parents who experience a stillbirth have a greater likelihood of relationship breakdown [44,48], and are more likely to neglect the emotional needs of siblings [45]. Studies have described significantly greater depression, anxiety and post-traumatic stress disorder after stillbirth [49,50], which extends into the subsequent pregnancy [51]. In addition, stillbirth also has a substantial negative emotional effect on healthcare professionals [52]. These wider costs are likely to impose a significant economic burden on the health care sector and society.

## Conclusions

In summary, this study has highlighted the paucity of evidence regarding the economic impact of stillbirth and the paucity of guidelines for the management of pregnancies following a stillbirth. Further research should systematically quantify the care involved following a stillbirth, in the next pregnancy and the time between the two events and ongoing effects of stillbirth. Notwithstanding the conservative nature of the financial estimates presented in this paper, stillbirth places a significant but unquantified financial burden on the NHS. The current costs associated with antenatal care in the next pregnancy (£15.1 million) combined with litigation costs (approximately £1.6 million) [53] imposes annual costs in the region of £16.7 million for the UK health service. Costs of investigations to ascertain the cause of stillbirth may contribute a significant part of this, but could work to reduce the antenatal care costs for the next pregnancy and for other women. Cost-effectiveness requires by definition a comparison of both costs and outcomes [39]. Clearly, the costs of this devastating outcome to pregnancy are far more than just those that can be estimated financially. In terms of effectiveness or outcomes, currently applied estimates of the number of quality adjusted life years (QALYs) for stillbirth are set at 25 and only take into account the life of the fetus (75 years discounted at 3.5% per year) [23]. Thus, the impact on quality of life to women and their families as a result of a stillbirth is so far un-quantified and unrepresented in cost-benefit analyses; further research is necessary to appropriately value these outcomes. This will allow the true economic impact of stillbirth to be presented alongside estimates for other perinatal complications including preterm birth and preeclampsia to facilitate appropriate prioritisation of resources [4-6]. Further evidence providing a clear understanding of the impact of stillbirth on costs and outcomes will facilitate the evaluation of the cost-effectiveness of interventions to prevent stillbirth in a realistic manner.

## Abbreviations

NHS: National Health Service; NICE: National Institute of Health and Clinical Excellence; RCOG: Royal College of Obstetricians and Gynaecologists.

## Competing interests

The authors declare that they have no competing interests.

## Authors’ contribution

TR and AH designed the study. HM and OV collected the cost data. HM carried out all the analysis with advice from AH and TR. HM and AH prepared the manuscript as lead authors. All authors contributed to the drafting of the manuscript and to the approval of the final manuscript. TR is the guarantor.

## Authors’ information

Hema Mistry and Alexander Heazell are joint lead authors.

## Pre-publication history

The pre-publication history for this paper can be accessed here:

http://www.biomedcentral.com/1471-2393/13/236/prepub
